# African potato (*Hypoxis hemerocallidea*): a systematic review of its chemistry, pharmacology and ethno medicinal properties

**DOI:** 10.1186/s12906-020-02956-x

**Published:** 2020-06-11

**Authors:** Celia M. J. Matyanga, Gene D. Morse, Mazuru Gundidza, Charles F. B. Nhachi

**Affiliations:** 1 Department of Clinical Pharmacology, College of Health Sciences, University of Zimbabwe, Harare, Zimbabwe; 2 School of Pharmacy, College of Health Sciences, University of Zimbabwe, Harare, Zimbabwe; 3 Center for Integrated Global Biomedical Sciences, School of Pharmacy and Pharmaceutical Sciences, University at Buffalo, Buffalo, NY USA; 4 Department of Pharmaceutical Technology, School of Industrial Sciences and Technology, Harare Institute of Technology, Belvedere, Harare, Zimbabwe

**Keywords:** African potato, Hypoxis hemerocallidea, Pharmacology, Pharmacokinetics, Chemistry, Traditional medicine

## Abstract

**Background:**

African Potato (*hypoxis hemerocallidea),* is used for enhancing immune system in Southern Africa. It is among the plants of intense commercial and scientific interest; hence, the aim of this study was to describe its chemistry and pharmacology.

**Methods:**

PubMed, Cochrane Controlled Trials Register (CENTRAL) and Google Scholar were searched independently for relevant literature. The last search occurred in October 2018. Other research material was obtained from Google. The following search terms were used, but not limited to: “African Potato”, “*hypoxis*”, “*hemerocallidea*”, “rooperol.” Articles that were explaining the chemistry and pharmacology of *hypoxis hemerocallidea* were included*.*

**Results:**

Thirty articles from PubMed, Cochrane and Google Scholar were eligible. Three webpages were included from Google. Results showed that the tuberous rootstock (corm) of African Potato is used traditionally to treat wasting diseases, testicular tumours, insanity, barrenness, impotency, bad dreams, intestinal parasites, urinary infection, cardiac disease and enhancing immunity. The plant contains hypoxoside, which is converted rapidly to a potent antioxidant, rooperol in the gut. The corm contains sterols, sterol glycosides, stanols, terpenoids, saponins, cardiac glycosides, tannins and reducing sugars. A dose of 15 mg/kg/day of hypoxoside is reportedly therapeutic. Preclinical studies of African Potato have shown immunomodulation, antioxidant, antinociceptive, hypoglycaemic, anti-inflammatory, anticonvulsant, antibacterial, uterolytic, antimotility, spasmolytic and anticholinergic effects. The common side effects of African Potato are nausea and vomiting, which subside over time. In vitro, African Potato demonstrated inhibitory effects on CYP1A2, 2C9, 2D6, 3A4, 3A5, CYP19-metabolism and induction of P-glycoprotein. In vivo, it did not alter the pharmacokinetics of efavirenz or lopinavir/ritonavir.

**Conclusion:**

African Potato is mainly used as an immunostimulant. The exact mechanisms of action for all the pharmacological actions are unknown. More research is required to substantiate claims regarding beneficial effects. There are many research gaps that require investigation including pharmacokinetic interactions with conventional drugs, especially those used in HIV/AIDS.

## Background

Medicines from natural sources have increased in popularity over orthodox medicines. Natural plants offer vast chemical diversity, which produce physiological changes in the human body [1]. In 2016, the worldwide annual market for herbal medicines was valued just above US$ 71 billion, and global health debates are focusing on traditional medicines [2]. Traditional medicines were used in historical eras and in populations in Africa, Asia, and Latin America and continue to be used due to cultural beliefs [3]. In the year 2002, severe acute respiratory syndrome (SARS) became a global disease outbreak, first appearing in China [4]. Many emergency measures were taken but there was no effective treatment [5]. The World Health Organization (WHO) reported that traditional medicine played a prominent role in the strategy to eradicate SARS in China. By late July 2003, no new cases were being reported [4].

Eighty percent of Africans use some form of traditional medicine [3] and the highest prevalence is among people living with HIV/ AIDS (PLWHA) [6, 7]. African Potato is one of the medicinal plants used for the management of human immunodeficiency virus (HIV) symptoms in Southern Africa. Its use in Africa is widespread and it is among the medicinal plants of intense commercial and scientific interest [8, 9].

African Potato, scientifically known as *hypoxis hemerocallidea* syn. *Hypoxis rooperi* belongs to the Hypoxidaceae family. Other common names include star lily, magic muthi or yellow stars [9]. The plant grows in the wild and is most prevalent in Southern Africa (mainly South Africa, Lesotho, Mozambique, and Zimbabwe). It is also found further into East Africa. The African Potato plant is easily identified by its star-shaped bright yellow flowers and green strap-like leaves. The tuberous rootstock (corm) is traditionally used to treat a wide variety of ailments. Extracts of the corm are used to make decoctions, which are taken as tonics against wasting diseases, tuberculosis, testicular tumors, other cancers, and HIV/ acquired immunodeficiency syndrome (AIDS) [10]. Traditionally, African Potato was used for insanity, barrenness, bad dreams, intestinal parasites, urinary infection and cardiac diseases among other diseases [11]. Nowadays it is used to increase immune function, for headache, dizziness, prostate hypertrophy, burns, and ulcers [10].

Albrecht, who thoroughly researched on African Potato, administered a methanolic extract of *H. hemerocallidea* to patients with HIV over 2 years in the mid-1990s. He reported that the CD4+ lymphocyte counts in these patients remained stable, while the serum p24 HIV antigen decreased and there was a decrease in expression of the HLA-DR CD8+ lymphocyte activation marker [12]. The HLA-DR CD8+ is used for identification of T lymphocytes and elevated levels are observed in HIV infection [13]. Albrecht concluded: “these studies have demonstrated that rooperol has potent, diverse and important pharmacological properties relevant to cancer, inflammation and HIV” [12].

The aim of this paper is to describe the chemistry, pharmacology and clinical properties of African Potato. Other objectives include identifying research areas for further study of the plant due to its widespread scientific interest. Reviewing the studies conducted on African Potato will reveal areas of further research.

## Methods

This systematic review adhered to the Preferred Reporting Items for Systematic Reviews and Meta-Analyses (PRISMA) guidelines [14]. A detailed literature review was conducted to describe the chemistry, pharmacology, clinical properties and pharmacologic claims made against African Potato.

### Identification of articles

The literature search was done using PubMed, Cochrane Controlled Trials Register (CENTRAL) and Google Scholar. These databases were searched independently for relevant literature through October 2018. The search was re-run on 16 May 2019 and no new studies were found. Other research material was obtained from open searches using Google. The following MeSH (Medical Subject Headings) terms and keywords were used, but not limited to: “African Potato” OR “hypoxis” OR “hemerocallidea” OR “rooperol”. An example of the search details in PubMed is given below: “African Potato”[All Fields] OR “hypoxis”[MeSH Terms] OR “hemerocallidea”[All Fields] OR “rooperol” (Supplementary Concept).

### Eligibility criteria

The material that described the chemistry, uses and pharmacology of *hypoxis hemerocallidea* were included*.* Other plant species were not included. The “sort by relevance” feature in Google Scholar was used; and where applicable, current articles and websites were selected for discussion. We did not restrict publication date. Clinical trials were included in the search. Table 1 shows the inclusion/ exclusion criteria.

**Table 1:** Inclusion/ exclusion criteria

Criteria	Inclusion	Exclusion
**Study design**	Clinical trial, quantitative, qualitative and mixed methods study, systematic/ narrative reviews	None
**Population**	All ages, all species	None
**Location**	Any country	None
**Date**	Studies available up to October 2018.	Studies published after October 2018
**Language**	English or translated to English	Not translated to English
**Research focus**	Describing the chemistry, uses and pharmacology of African potato (*hypoxis* genus)	Describing other plant species or genus
**Document type**	Full text article of research articles, clinical trials, systematic reviews, scientific reports, ethnopharmacological studies, ethnobotanical surveys, commentaries, case reports, conference proceedings.	Full text of document not available

## Results and discussion

**Fig. 1:**
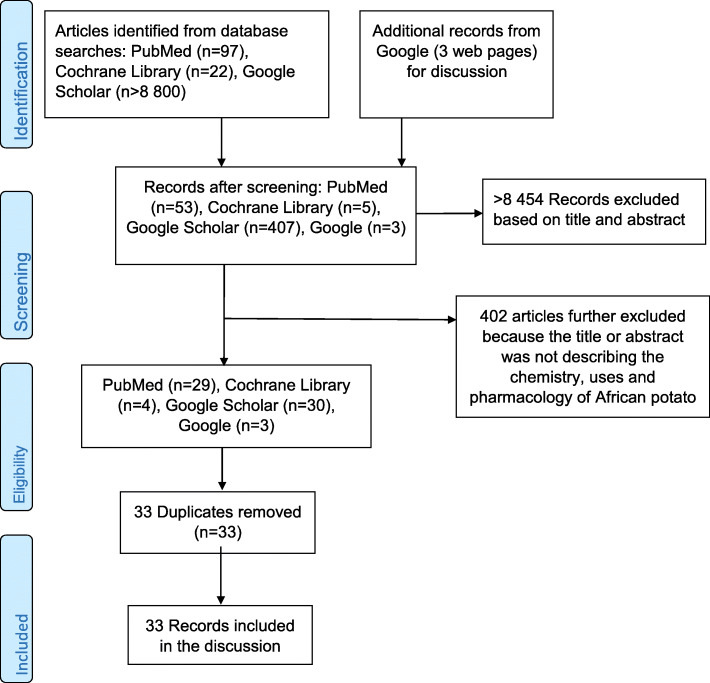
PRISMA flow chart

Thirty-three articles were used for data collection. Figure 1 shows the flow diagram for the data collection. The general characteristics of the articles and the data extracted are shown in Table 2.

**Table 2:** General characteristics of included studies

Study number	Study design	Author, year [reference]	Data extracted
1.	Clinical trial	Albrecht et al., 1995 [15]	Safety of β-sitosterol; dose and metabolic pathway of hypoxoside
2.		Donald et al., 1997 [16]	Pharmacological effects of phytosterols
3.		Mogatle et al., 2008 [17]	African potato drug interactions
4.		Gwaza et al., 2013 [18]	African potato drug interactions
5.		Berges et al., 1995 [19]	Clinical properties, the dosage of β-sitosterol
6.	Pilot study, open-label intervention	Bouic et al., 2001 [20]	Pharmacological effects of β-sitosterol
7.	Quantitative, experimental	Boukes GJ et al., 2010 [21]	Pharmacology
8.		Awad et al., 2000 [22]	Mechanism of action
9.		Bouic et al., 1996 [23]	Pharmacology and bioavailability of β-sitosterol
10.		Lamprecht et al., 2000 [24]	β-sitosterol and the glucoside mixture improving CD4 count
11.		Albrecht et al., 1995 [25]	Mechanism of action, metabolism and pharmacokinetics of hypoxoside
12.		Kruger et al., 1994 [26]	Metabolism of hypoxoside
13.		Nair et al. 2007, [27]	Metabolism of African potato
14.		Gwaza et al., 2009 [28]	Drug interactions of *hypoxis* extracts
15–22.	Experimental preclinical (in vivo)	As shown in Table 3	Pharmacologic activities of Africa Potato in different species
23.	Qualitative screening	Zimudzi C, 2014 [1]	Chemistry
24.		Nair et al., 2006 [37]	Dosage of African potato
25.	Systematic review	Ncube et al., 2013 [38]	Uses of African potato and dosage forms (and strengths) available
26.	Narrative review	Drewes SE et al., 2008 [10]	Chemistry, ethnopharmacological properties
27.		Mills E et al., 2005 [11]	Pharmacology, chemistry, ethnopharmacological properties
28.		Bouic 2001, [39]	Chemistry and pharmacological uses
29.		Ling et al.,1995 [40]	Pharmacological uses, mechanism of action of phytosterols
30.		Saeidnia et al., 2014 [41]	Pharmacological effects
31.	Not applicable - Website	Natures Health website, [42]	Dosage of African potato capsules
32.		Green Herbs & Nutrition’s Stores website, [43]	Dosage of African potato capsules
33.		Puer Orijins catalogue, [44]	Other dosage forms available

### Pharmacology and chemistry

*Hypoxis* species have been reported to produce a variety of phytoglycosides; extensive research has been focused on the norlignan diglycoside hypoxoside and its aglycon rooperol. The main glycoside that is isolated from the *Hypoxis* spp. is hypoxoside [21]. Following oral administration, hypoxoside is metabolized in the gut to rooperol by β-glucosidase. There are two glucose units at the ends of two benzene rings on hypoxoside [10]. These units are oxidized by β-glucosidase to form the aglycone, rooperol. The enzyme β-glucosidase is found mostly in the gastrointestinal tract (GIT) and is released by rapidly dividing cancer cells. Rooperol is the biologically active compound that is associated with claims of medicinal properties [11].

Other constituents in *hypoxis* include various sterols and their glycosides, and these may have biological importance. *H. hemerocallidea* contains β-sitosterol (BSS), β-sitosterol glucoside (BSSG), campesterol and stigmasterol [39]. These plant sterols (phytosterols) have biologic roles in animal and human health. Phytosterols are incorporated into functional foods and inhibit the absorption of cholesterol from the diet. They also have prophylactic and therapeutic uses in hypercholesterolemia, cardiovascular disease and atherosclerosis [40].

Among the phytosterols, β-sitosterol and its glycoside have been studied most for their pharmacological effects [41]. In vitro, the combination of BSS and BSSG indicated anti-inflammatory effects mediated by the inhibition of interleukin 6 and tumor necrosis factor secretion. The anti-inflammatory effects of the mixture relieved rheumatoid arthritis in humans. Another small pilot study reported that the BSS/ BSSG mixture resulted in significant improvement in allergic rhinitis/sinusitis after 12 weeks and this was attributed to immunological changes in the cytokine profiles produced by lymphocytes [40]. In vitro, phytosterols can affect different levels of tumor development and they have immune-modulating properties [41]. Phytosterols initiated programmed cell death (apoptosis) in human colon cancer, breast cancer, and prostate cancer. The probable mechanism was the activation of the protein phosphatase A2 pathway and the sphingomyelin cycle [22].

Rat models suggest that phytosterols may offer protection against breast, colon and prostate cancer [39]. In Phase I clinical trials, BSS has proven to be safe [15]. Sitosterols are poorly absorbed from the gastrointestinal tract. In humans, oral bioavailability is no more than 5% and it is 9% in dogs [23]. However, with advanced formulation technology many targeted drug delivery systems may provide alternative approaches for compounds with low bioavailability [45]. If successful, targeted delivery systems could aid in the delivery of phytosterols to facilitate clinical trials. An important knowledge gap is the drug interactions that may occur in immunocompromised patients who require many other medications (polypharmacy).

In another study, domestic cats were infected with a model of HIV. Cats treated with phytosterols maintained stable CD4 cell counts compared to placebo; the mortality between the two groups was significantly different [24]. In humans, an open-label study compared the efficacy of BSS/ BSSG with placebo in HIV infected treatment-naïve patients. During the time of the study, antiretroviral treatment (ART) was not affordable to most patients. Within 12 months, patients with > 500 CD4 cells/ μl at baseline maintained their CD4 cell count and plasma viral loads were significantly decreased. Those with advanced HIV at baseline (< 200 CD4 cells/ μl) still had disease progression. Patients in the BSS/ BSSG arm maintained a favorable T_H1_ response and their cell-mediated immunity was likely to be responsible for their response [20]. These findings concur with clinical trials that were conducted later that early initiation of ART delays the time to AIDS events [46]. In addition, there should be more research on herbs that enhance immune function in immunocompromised individuals to slow the progression of the disease. Again, due to polypharmacy, possible drug-herb interactions should be considered. Phytosterols are associated with faster clinical recovery in pulmonary tuberculosis [16] and possess anti-inflammatory, wound healing, analgesic, anti-helminthic, anti-mutagenic, anti-oxidant, neuroprotective and anti-diabetic properties [41].

There is limited knowledge on other secondary metabolites of *hypoxis*. As of 17 October 2018, a literature search found one study in Zimbabwe that compared the phytochemical profiles and cytotoxicity of four species of *hypoxis*. These were *H. hemerocallidea, H. rigidula, H. galpinii and H. obtuse.* Although this study did not quantify the phytochemicals, corm extracts of all four species indicated the presence of terpenoids, saponins, cardiac glycosides, tannins and reducing sugars. All species screened negative for alkaloids, flavonoids, and anthraquinones [1]. In other plant species, these phytochemicals are claimed to have curative activity against several pathogens [47]. The phytochemicals identified in this study can be attributed to the biologic activities of *hypoxis*. Terpenoids have antimicrobial and antioxidant properties and they are explored as cytotoxic and antineoplastic agents [48]. Saponins from plant sources have various pharmacologic effects like antimicrobial, anticancer, anthelmintic, antioxidant, antidiabetic, anticonvulsant, analgesic, antispasmodic, hypocholesterolemic, antitussive and cytotoxic activities [49]. Cardiac glycosides inhibit the Na^+^/K^+^ pump thus slow the heart rate and increase the contractility of the heart muscle. Although they improve the cardiac output and heart function, their use is associated with toxicity because of a narrow therapeutic index [50]. Tannins have anti-oxidative activities; due to these properties, they are anti-carcinogenic and anti-mutagenic. In addition, tannins have antimicrobial properties, accelerate blood clotting, reduce blood pressure, decrease serum lipid levels and modulate immune responses [51]. Reducing sugars have a regulatory role in plants, controlling their growth and development to provide resistance against diseases [52].

It is well known that combining several bioactive compounds result in a greater pharmacological response than using the single components [53]. With traditional medicines, isolating the desired phytochemicals and combining them can result in achieving the desired pharmacological response. More laboratory and clinical studies with *hypoxis* are required in this area of research.

### Preclinical pharmacologic activities (Table 3)

#### Absorption and metabolism

**Fig. 2:**
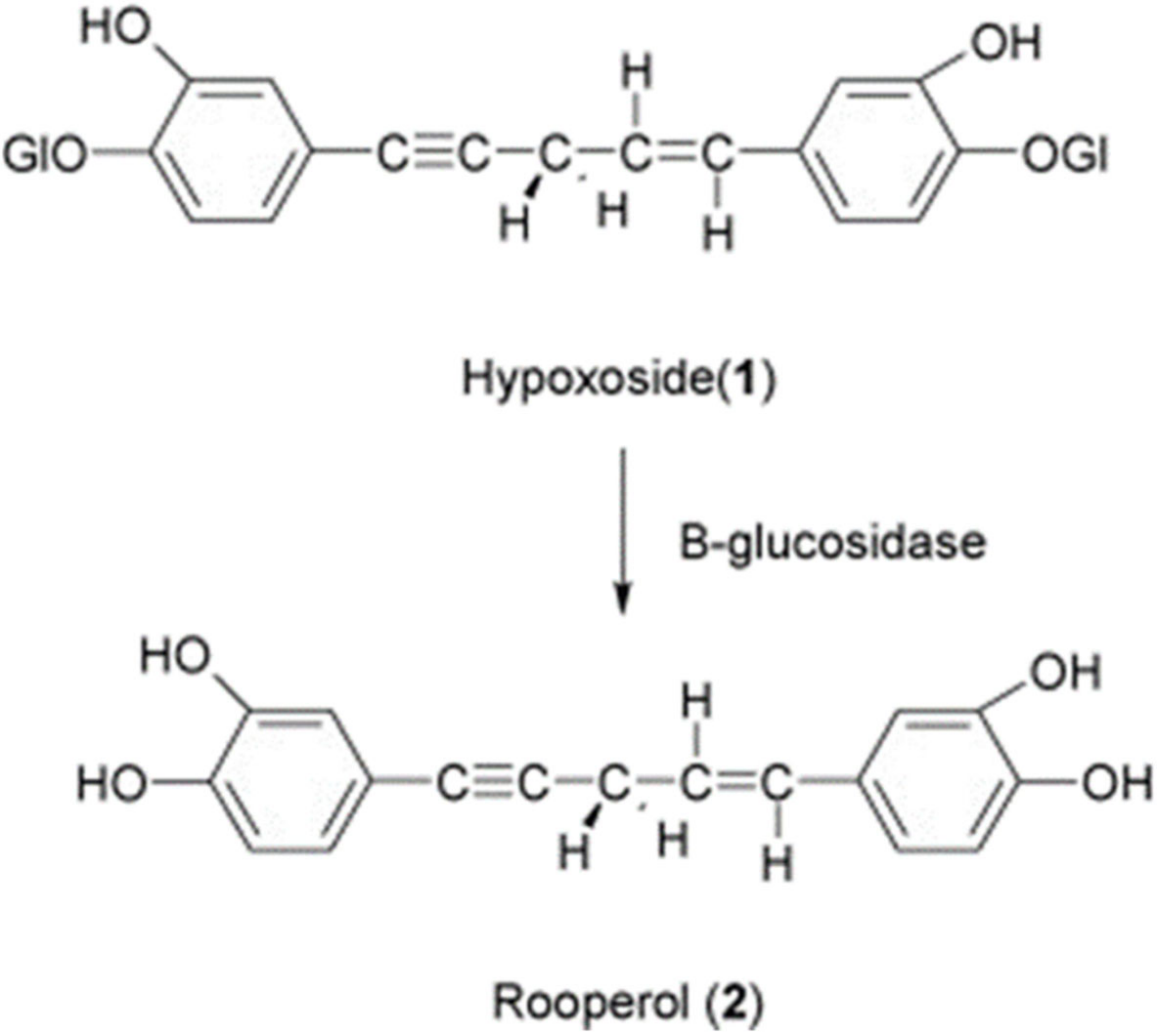
Structures of hypoxoside and rooperol [10]

**Fig. 3:**
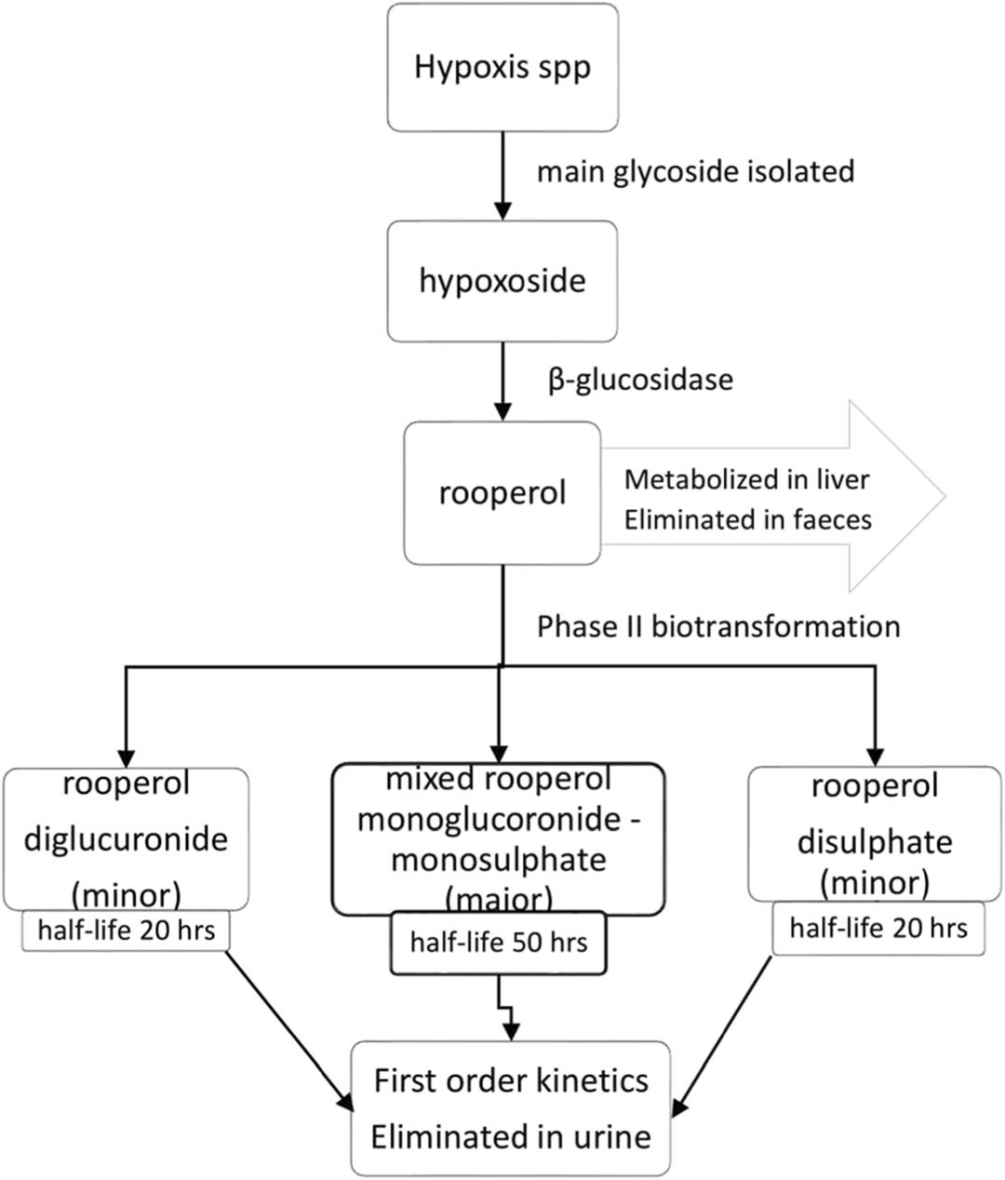
Metabolic Pathway of African Potato in Humans [11, 23]

After oral administration, hypoxoside is not absorbed and undergoes enzymatic hydrolysis. In the circulatory system, hypoxoside is converted to rooperol (Fig. 2) by β-glucosidase. Intragastric administration of hypoxoside in mice resulted in deconjugation by bacterial β-glucosidase to form rooperol in the colon. In mice, neither hypoxoside nor rooperol metabolites were detectable in the blood. There were only Phase II metabolites of sulphates and glucuronides present in the bile of mice, rats, and dogs [25]. However, in humans and baboons, these metabolites appear in the plasma at relatively high concentrations [26]. The end products of the hydrolysis were rooperol, dehydroxyrooperol and bis-dehydroxyrooperol [15]. The metabolic pathway of African Potato is illustrated in Fig. 3.

**Table 3:** Preclinical (in vivo) Pharmacologic Activities of Africa Potato in different formulations

Species	Dose and administration	Parameters assessed	Conclusions	Reference
Male rats	Acute testing: 0.45; 0.90 and 1.8 mg/ kg infusion	Urine volume and total urinary outputs of creatinine, sodium, and potassium.	Increased plasma creatinine concentration, renal fluid, and electrolyte retention and reduced GFR compared with controls, APE may impair renal function.	[29]
	Chronic: APE 30 mg/ kg infusion			
Healthy mice	Corm aqueous extract (100–800 mg/kg i.p.)	Effect against pentylenetetrazole-, picrotoxin- and bicuculline-induced seizures.	APE has anticonvulsant activity possibly by enhancing GABAergic neurotransmission and/or action in the brain.	[30]
			Doses of ≥400 mg/kg resulted in dose-related sedation and drowsiness.	
		Phenobarbitone and diazepam used as the reference.		
Rats and guinea-pigs	Corm aqueous extract 25–400 mg/ml orally	Uterine horns isolated from rats and guinea-pigs.	Extract showed uterolytic activity	[31]
			Inhibited the amplitude and sometimes, the frequency of the spontaneous, rhythmic contractions.	
			Relaxed pregnant uterine muscles.	
			Mechanism is unknown, probably mediated through a non-specific spasmolytic mechanism.	
			Extracts to 2.5 g/kg did not produce any toxic manifestations or mortalities.	
Newborn suckling rats	African Potato ethanol or aqueous extract (50 mg/kg and a high dose of 200 mg/kg) via a stomach tube	Viscera removed for gross and microscopic morphometric measurements.	At a low dose, the mean weight gain was significantly increased.	[32]
			The high dose of aqueous extract increased the weight of the caeca.	
			The low dose of alcohol extract reduced the pancreas weight.	
			No adverse effects, no signs of pathology.	
Healthy rats and mice	Corm aqueous extract (APE, 50–400 mg/kg, orally)	Effect against castor oil-induced diarrhea, entero-pooling, intestinal transit, and intestinal fluid.	APE delayed the onset of copious diarrhea, reduced number, and weight of wet stools, inhibited the severity of diarrhea, inhibited intestinal transit and delayed gastric emptying.	[33]
		Atropine and loperamide used as positive controls.		
			Speculated mechanism that the sterols, stanols and sterolins, especially rooperol and β-sitosterol are responsible for antimotility, spasmolytic and anticholinergic effects.	
Healthy mice	AP methanolic extract (15 mg of extract orally)	After *Brachyspira hyodysenteriae* –induced typhlocolitis; weight loss, gross and histological lesions, MPO activity, and intestinal epithelial proliferation were evaluated.	AP extract reduced weight loss, the severity of typhlocolitis, inflammation and intestinal epithelial proliferation.	[34]
Albino rats	Aqueous corm decoction (10 ml/kg) and 20 ml/kg orally	Parameters assayed were TBARS, SGOT, SGPT, GSH, ascorbic acid, tocopherol, superoxide dismutase and glutathione peroxidase in RBC and in the liver.	Protection from oxidative stress generated by chloroquine, strengthen the antioxidant system under normal conditions.	[35]
STZ – Induced diabetic male Wistar rats	Aqueous solution (200 mg/kg or 800 mg/kg) administered orally	Oxidative stress biomarkers, hepatic injury, and selected biomarkers in the liver and kidney.	Both dosages showed significant antihyperglycemic effects, both showed antioxidant effects in the liver tissue.	[36]
			At higher concentration, the activity of liver enzymes was increased and a negative effect on the kidneys was observed.	
			Lower concentrations ameliorated liver injury.	

*AP* African Potato, *APE* African Potato aqueous extract, *i.p.* Intraperitoneal, *GFR* glomerular filtration rate, *GSH* reduced glutathione, *MPO* myeloperoxidase, *RBC* red blood cells, *SGOT* serum glutamate oxaloacetate transaminase, *SGPT* serum glutamate pyruvate transaminase, *STZ* streptozotocin, *TBARS* thiobarbituric acid reactive substance

The presence of rooperol was analyzed in faeces and urine in humans. After administration of 1 g of hypoxoside, rooperol was present in faeces at 6-h post-dosing. No rooperol was detected in urine after 24 h. Some of the rooperol was absorbed from the colon and some were eliminated in the faeces. The formation and absorption of rooperol was a zero-order saturable process [15].

#### Drug interaction studies

The effects on cytochrome P450 (CYP) - mediated metabolism of African Potato were studied in vitro using cell lines. The African Potato extracts demonstrated inhibitory effects on CYP3A4-, 3A5- and CYP19-mediated metabolism and high induction of P-glycoprotein (P-gp) as compared to ritonavir, the positive control [27]. Another study evaluated the effect of *hypoxis* on drug interactions in vitro using human liver microsomes. In methanol extracts, at least 95% inhibitory effects were observed for CYP1A2, 2C9, 3A4 and 2D6 compared to positive controls. Aqueous hypoxis extracts led to moderate CYP inhibition. The extracts of hypoxis indicated no significant inhibition of P-gp although the authors suggested some effect on P-gp was possible at higher concentrations than those used in the assays [28].

These in vitro results served as the foundation for in vivo interaction studies for African Potato. A study conducted in South Africa determined the effect of African Potato on efavirenz pharmacokinetics. Ten healthy volunteers participated in this single-dose, two-phase sequential study over 31 days [17]. Efavirenz is a non-nucleoside reverse transcriptase inhibitor (NNRTI) effective against HIV-1. It is the backbone of combination antiretroviral therapy (cART) in Africa and is mainly metabolized by CYP2B6 and to a lesser extent CYP3A4 [54]. For the South African study, the following parameters were used to determine interactions: AUC_0–48_, C_max_, T_max_, T_1/2,_ and K_el_. The results indicated that the 90% confidence intervals (CI) for C_max_ and AUC_0–48_ were within the limits of 80–125% interval. Thus, the investigators concluded that the African Potato did not alter efavirenz pharmacokinetics. The investigators recommended that further research is needed to investigate African Potato and other antiretrovirals especially those that are P-gp substrates or CYP3A4 metabolites [17]. Although this study had a clear and concise methodology, the sample size calculations were not well explained, especially considering the intra-individual variability of the AUC and C_max_ for efavirenz. In addition, single-dose studies do not consider induction that occurs during chronic dosing.

Another study investigated the effect of African Potato on the steady-state pharmacokinetics of ritonavir-boosted lopinavir (LPV/r). Lopinavir/ ritonavir is a potent HIV protease inhibitor combination that is used with other antiretrovirals for the treatment of HIV infection. Lopinavir (LPV) increases ritonavir (RTV) concentrations through inhibition of CYP3A4. LPV is metabolized primarily by hepatic and gastrointestinal CYP3A4. They hypothesized that since in vitro studies indicate that extracts of African Potato have a significant inhibitory effect on CYP3A4 this could lead to an increase in exposure, associated with an increased cholesterol/ diabetes risk. This study was an open-label, two-period; fixed sequence, crossover pharmacokinetic drug interaction study. Sixteen healthy, HIV-seronegative adult volunteers between 18 to 60 years were enrolled. The following parameters were used to determine interactions: AUC_0–18_, C_max_, C_trough_, T_max_, T_1/2_, CL_F_ and K_el_. Results indicated that steady-state plasma concentration-time profiles of LPV with and without African Potato were similar as reflected by the 90% confidence intervals that were within the 80–125% limit. The effect on ritonavir was not analyzed in this study. Total cholesterol and triglycerides were elevated but within limits during LPV/r treatment [18]. The investigators concluded that African Potato had no significant effect on the steady-state pharmacokinetics of LPV. This study was well designed although the results cannot be generalized to other populations. It would have been ideal to use participants from Africa, where African Potato use is prevalent. Clinical studies involving African Potato or its constituents are summarized in Table 4.

**Table 4:** Clinical Studies involving African Potato and/ its Constituents

Population	Study Design	Dose and administration	Parameters Assessed	Major Findings	Reference
16 (12 males, 4 females) healthy HIV-seronegative adults, USA. The median age (range) was 28 yrs. (19–53 yrs). Median weight (range) was 78 kg (53–96 kg)	An open-label, two-period, fixed sequence, cross-over pharmacokinetic drug interaction study.	LPV/r 400/100 mg tablet orally twice a day for 14 days, then LPV/r with African potato (15 mg/ kg hypoxoside) orally once daily for 7 days.	Plasma samples collected on day 14 after LPV/r alone and day 21 after LPV/r plus African potato for AUC, C_max_, C_trough_, T_max_, T_1/2_, K_el_, CL_F_. Time of collection: within 1 h before dosing and at 1, 2, 4, 6, 8, and 12 h post-dose for both phases.	African potato combined with LPV/r was not associated with any change in LPV/r AUC, C_max_, C_trough_. No serious adverse events observed.	[18]
10, (9 black, 1 white) healthy HIV-negative males. The mean age (range) was 23 yrs. (19–27 yrs).	A single-dose, two-phase sequential study.	Efavirenz 600 mg tablet orally on day 1, then from day 16, a traditionally prepared African Potato decoction (15 mg/ kg of hypoxoside) given daily until day 30. On day 28, efavirenz 600 mg tablet was given orally.	Phase 1 started on day 1 and phase 2 on day 29, each phase lasting 3 days. Plasma samples were collected before dosing and at 0.5, 1, 1.5, 2, 2.5, 3, 3.5, 4, 5, 6, 8, 12, 18, 24, 36 and 48 h after dosing. Samples were assayed for AUC, C_max_, T_max_, T_1/2_ and K_el_.	The geometric mean ratios of C_max_ and AUC were within the limits of 80–125%. African potato did not alter the pharmacokinetics of efavirenz. No serious adverse effects were noted.	[17]
37 adult male patients with PTB in South Africa. 19 in the active group (mean age 43 yrs. and weight 49 ± 6 kg) and 18 in the placebo group (mean age 37 yrs. and weight 51 ± 9 kg).	A double-blinded, randomized, placebo-controlled trial to evaluate the effects of BSSG and BSS in the treatment of PTB.	Randomized to receive either the active capsule with sitosterols (0.2 mg BSSG, 20 mg BSS, 200 mg talcum) or placebo (200 mg talcum). One capsule three times daily together with their standard antituberculosis regimen (isoniazid, rifampicin, pyrazinamide) for 6 months.	Sputum culture positivity, chest radiography, weight gain, Mantoux test response, routine hematology and liver function. PTB was confirmed by sputum smear microscopy for acid-fast bacilli and culture for *Mycobacterium tuberculosis*.	Compared to placebo, there was significant weight gain, higher lymphocyte and eosinophil counts in PTB patients receiving sitosterols in addition to antituberculosis therapy.	[16]
24 patients with histologically proven squamous, large-cell, or adenocarcinoma, South Africa.	A randomized, open, single-dose study of the pharmacokinetic behavior of hypoxoside in patients with lung cancer.	Three groups with dosage levels of 1600, or 2400, or 3200 mg standardized *hypoxis* plant extract (200 mg capsules). The first 6 patients in the multiple-dose study took 4 capsules 3 times daily for 11 days.	Serum samples were collected at regular intervals up to 75 h after single doses for the detection of hypoxoside metabolites. In the multiple-dose study, blood was drawn before the first dose each day. Pharmacokinetic parameters of the major metabolites were analyzed using different models in the NONMEM program.	After oral ingestion, hypoxoside undergoes complete Phase II biotransformation to diglucuronide, disulphate, and mixed glucuronide-sulphate metabolites. Neither hypoxoside nor rooperol appears in the blood. The half-lives of the major metabolite were 50 h and 20 h for the minor metabolites.	[15]
200 male patients with symptomatic BPH not on any treatment, Germany.	A randomized, double-blind, placebo-controlled multicenter study.	Randomized to receive either 20 mg BSS capsule (including 01 mg BSSG) three times per day or placebo.	The endpoints were a difference of modified Boyarsky score (recorded monthly) for 6 months, changes in IPSS, urine flow, and prostate volume (every 3 months.)	There were improvements in the modified Boyarsky score, symptoms with the IPSS questionnaire, quality of life score and urinary flow in the BSS group compared to placebo. BSS was shown to be effective in the treatment of BPH.	[19]

*AUC* area under the concentration-time curve within a dosing interval, *BPH* benign prostatic hyperplasia, *BSS* β-sitosterol, *BSSG* β-sitosterol glucoside, *C*_*max*_ maximum concentration following dose administration, *C*_*trough*_ plasma concentration at the end of the dosing interval, *CL*_*F*_ apparent clearance, *IPSS* International Prostate Symptom Score, *K*_*el*_ elimination rate constant, *LPV/r* ritonavir-boosted lopinavir, *PTB* pulmonary tuberculosis, *T*_*max*_ time to reach C_max_, T_1/2_ – half-life

Both the South African and USA studies were testing African Potato in healthy individuals. Literature reveals that African Potato is widely used for its immune-enhancing properties in HIV infected individuals [11]. Since African Potato has shown to be safe and well tolerated in healthy individuals, further research should focus on people living with HIV/AIDS. It would also be necessary to study the interactions of African Potato in HIV infected individuals taking other antiretroviral drugs.

#### Dosage recommendations

Traditionally, African Potato is cut into cubes or shredded and boiled in water for 20 min before the decoction is consumed orally. A survey conducted among traditional healers in South Africa was used to calculate the dose of African Potato. An average of about 20 g of freshly shredded African potato boiled in 250 mL of water was prescribed for daily consumption. African potato was mainly prescribed to boost immunity [37].

For the treatment of benign prostatic hyperplasia, African Potato dosed at 20 mg of β-sitosterol three times a day was found to be therapeutic [19]. According to literature, an oral dose of 15 mg/ kg/ day is reportedly therapeutic [18]; however, it is unknown whether this dose is effective for all the claims against African Potato. Other sources state that 2400 mg taken daily is therapeutic [15]. Standardized capsules are available online which contain from 300 to 350 mg *hypoxis hemerocallidea*. The doses for these formulations vary; some stating one capsule twice daily and some stating two tablets 3 times a day for the first 5 days, then one tablet 3 times a day [42, 43]. In South Africa, herbal formulations of African Potato are mainly used to enhance the immune system. The herbal formulations are available as capsules, tonics, creams and tinctures containing 300–500 mg *hypoxis hemerocallidea* or sterols/ sterolins [38]. With the many claims against the plant, it is unknown if this dose is a standard dose. Besides capsules, other formulations available include powders, face creams, night cream, nasal spray, soap, tissue oil, toner and exfoliator [44]. There is a knowledge gap in the therapeutic dosage for herbal medicines since most of the recommended doses are based on anecdotal information [11]. Furthermore, there is limited research in clinical trials using herbal medicines [55]. This is an area of research that could be explored further, even with African Potato.

## Conclusion

African Potato rootstock (corm) is used to treat a wide variety of ailments. It is mainly used as an immunostimulant in people living with HIV/ AIDS. The active components include rooperol, which is an antioxidant and several phytosterols. The mechanisms of action for all the pharmacological actions are unknown. Some of the pharmacological actions were reported in older studies and there is a need for studies to substantiate the claims using current technology and with the application of systems pharmacology. African Potato is of intense commercial and scientific interest and more clinical trials should be performed to evaluate dosage regimens. The plant shows a good safety profile although there are no studies that have demonstrated safety in children, pregnant and lactating women. More research is required to substantiate the many claims that recommend the use of African Potato. There are important research gaps on the possible interactions with conventional drugs, especially those used in HIV/AIDS.

## Supplementary information

**Additional file 1.** PRISMA 2009 checklist.

## Data Availability

All data generated and reviewed during this study was included in this manuscript and in the tables and figures.

## Abbreviations

AIDS: Acquired immunodeficiency syndrome
AP: African Potato
APE: African Potato aqueous extract
ART: Antiretroviral treatment
AUC: Area under the concentration-time curve within a dosing interval
BSS: β-sitosterol
BSSG: β-sitosterol glucoside
cART: Combination antiretroviral therapy
CL_F_: Apparent clearance
C_max_: Maximum concentration following dose administration
C_trough_: Plasma concentration at the end of the dosing interval
CYP: Cytochrome P450
GFR: Glomerular filtration rate
GIT: Gastrointestinal tract
GSH: Reduced glutathione
K_el_: Elimination rate constant
HIV: Human immunodeficiency virus
i.p: Intraperitoneal
LPV/r: Ritonavir-boosted lopinavir
LPV: Lopinavir
MPO: Myeloperoxidase
NNRTI: Non-nucleoside reverse transcriptase inhibitor
P-gp: P-glycoprotein
PLWHA: People living with HIV/ AIDS
PRISMA: Preferred Reporting Items for Systematic Reviews and Meta-Analyses
RBC: Red blood cells
RTV: Ritonavir
SARS: Severe acute respiratory syndrome
SGOT: Serum glutamate oxaloacetate transaminase
SGPT: Serum glutamate pyruvate transaminase
STZ: Streptozotocin
TBARS: Thiobarbituric acid reactive substance
T_max_: Time to reach C_max_
T_1/2_: Half-life
WHO: World Health Organization
